# Formation model of anxiety disorders in internally displaced persons in war context in Ukraine

**DOI:** 10.1192/j.eurpsy.2025.1022

**Published:** 2025-08-26

**Authors:** H. Kozhyna, K. Zelenska, L. Gaichuk, S. Isaenko, A. Nartova

**Affiliations:** 1 Department of Psychiatry, Narcology, Medical Psychology and Social Work, Kharkiv National Medical University, Kharkiv; 2 Mental Health Clinic by Dr.Isaenko, Kyiv, Ukraine

## Abstract

**Introduction:**

Since the beginning of the full-scale war with Russia, 7.7 million Ukrainians have been forcibly displaced from their homes and are currently living in internally displaced persons (IDP) camps. According to the International Organization for Migration (IOM), the proportion of IDPs in Ukraine’s total population has reached 17.5%, indicating that one in six individuals has been displaced.

**Objectives:**

The purpose of the study is to investigate the clinical, psychological and pathological patterns of anxiety disorders of psychogenic genesis. The main group consisted of 93 IDP patients with anxiety disorders, both sexes, aged 20-55 years.

**Methods:**

SCL-90-R; HAM-A, HAM-D; Spielberger-Khanin Scale and Questionnaire of neuropsychological stress by T.A. Nemchin.

**Results:**

It has been established that the clinical structure of anxiety disorders in the examined patients is represented by a mixed anxiety-depressive reaction (28.2±1.2% of the examined), panic disorder (36.4±1.3%) and generalised anxiety disorder (35.4±1.3%). At the same time, in the clinical structure of anxiety disorders in IDPs, along with the dominance of anxiety symptoms, there is a high severity of depressive manifestations. Based on data obtained in the course of our research, a multifactorial model of formation of anxiety disorders in IDPs was developed. The catalyst for anxiety disorders is the very fact of forced displacement, uncertainty of future, situations of loss, effects of combat stress, information stress and situations of increased responsibility.

High levels of anxiety, somatisation, depression, obsessive-compulsive disorders, interpersonal sensitivity, and phobic anxiety according to SCL-90-R scale are prognostically significant in formation of anxiety disorders; severe depressive and anxiety episodes according to the Hamilton Anxiety and Depression Scales; high levels of personal and situational anxiety according to the Spielberger-Khanin Scale and excessive levels of neuropsychological stress.

**Conclusions:**

The basis for the formation of anxiety disorders in IDPs is a low level of resilience, which entails a high level of social frustration in this cohort. We have developed and tested a comprehensive personalised system for treatment of anxiety disorders in IDPs with differentiated use of psychopharmacotherapy, psychotherapy and psychoeducation.

**Disclosure of Interest:**

None Declared

